# “Burning mouth syndrome in Parkinson’s disease: dopamine as cure or cause?” Letter to the Editor Reply

**DOI:** 10.1007/s10194-012-0487-9

**Published:** 2012-10-05

**Authors:** Elizabeth A. Coon, Ruple S. Laughlin

**Affiliations:** Neuromuscular Division, Department of Neurology, Mayo Clinic, Rochester, 55905 MN USA

We appreciate Drs. Fortuna and Pollio’s interest in our case report describing a woman with Parkinson’s disease who developed burning mouth syndrome (BMS) with carbidopa/levodopa therapy that resolved after its discontinuation and initiation of a dopamine agonist.

The authors bring up an excellent and also contentious point regarding the semantics of burning mouth syndrome [1, 2]. We utilize the current diagnostic criteria from the International Headache Society which classifies primary BMS under “Central causes of facial pain” [3]. The intent of reporting our case was not to define the syndrome, but instead to contribute anecdotal evidence to the hypothesis that the pathophysiology of BMS is related to dopamine dysregulation. We believe our case supports this notion given our patient’s diagnosis of Parkinson’s disease and apparent accentuation of BMS symptoms with administration of carbidopa/levodopa [4–6]. Our speculation is that carbidopa/levodopa’s central action led to the neurophysiological manifestations of BMS. Beyond superficially dismissing our patient’s symptoms as drug induced, the value in our case is related to deeper consideration of the underlying pathogenesis of BMS.

Although we agree with the authors that it is quite difficult to delineate the role of depression and anxiety in cases of burning mouth syndrome [7], we did not think these psychiatric factors were of primary relevance in our case as there was no history of such. As outlined in our report, her BMS symptoms precisely correlated to carbidopa/levodopa administration.

We appreciate the input of Drs. Fortuna and Pollio and come to the agreement that it is helpful to look at BMS from a multidisciplinary perspective. This approach will only advance our understanding of a disease plaguing patients who seek care from a variety of specialists.
